# A comprehensive review of the lipid cubic phase or *in meso* method for crystallizing membrane and soluble proteins and complexes

**DOI:** 10.1107/S2053230X14026843

**Published:** 2015-01-01

**Authors:** Martin Caffrey

**Affiliations:** a Membrane Structural and Functional Biology Group, School of Medicine and School of Biochemistry and Immunology, Trinity College Dublin, Dublin, Ireland

**Keywords:** crystallization, lipid cubic phase, macromolecular crystallography, membrane-protein structure, mesophase, robot, structure–function, water-soluble proteins

## Abstract

The lipid cubic phase or *in meso* method is a robust approach for crystallizing membrane proteins for structure determination. The uptake of the method is such that it is experiencing what can only be described as explosive growth. This timely, comprehensive and up-to-date review introduces the reader to the practice of *in meso* crystallogenesis, to the associated challenges and to their solutions. A model of how crystallization comes about mechanistically is presented for a more rational approach to crystallization. The possible involvement of the lamellar and inverted hexagonal phases in crystallogenesis and the application of the method to water-soluble, monotopic and lipid-anchored proteins are addressed. How to set up trials manually and automatically with a robot is introduced with reference to open-access online videos that provide a practical guide to all aspects of the method. These range from protein reconstitution to crystal harvesting from the hosting mesophase, which is noted for its viscosity and stickiness. The sponge phase, as an alternative medium in which to perform crystallization, is described. The compatibility of the method with additive lipids, detergents, precipitant-screen components and materials carried along with the protein such as denaturants and reducing agents is considered. The powerful host and additive lipid-screening strategies are described along with how samples that have low protein concentration and cell-free expressed protein can be used. Assaying the protein reconstituted in the bilayer of the cubic phase for function is an important element of quality control and is detailed. Host lipid design for crystallization at low temperatures and for large proteins and complexes is outlined. Experimental phasing by heavy-atom derivatization, soaking or co-crystallization is routine and the approaches that have been implemented to date are described. An overview and a breakdown by family and function of the close to 200 published structures that have been obtained using *in meso*-grown crystals are given. Recommendations for conducting the screening process to give a more productive outcome are summarized. The fact that the *in meso* method also works with soluble proteins should not be overlooked. Recent applications of the method for *in situ* serial crystallography at X-ray free-electron lasers and synchrotrons are described. The review ends with a view to the future and to the bright prospects for the method, which continues to contribute to our understanding of the molecular mechanisms of some of nature’s most valued proteinaceous robots.

## Abstract 2

A comprehensive and up-to-date review of the lipid cubic phase or *in meso* method for crystallizing membrane and soluble proteins and complexes is reported. Recent applications of the method for *in situ* serial crystallography at X-ray free-electron lasers and synchrotrons are described.

## 1. Introduction   

**Figure 1:**
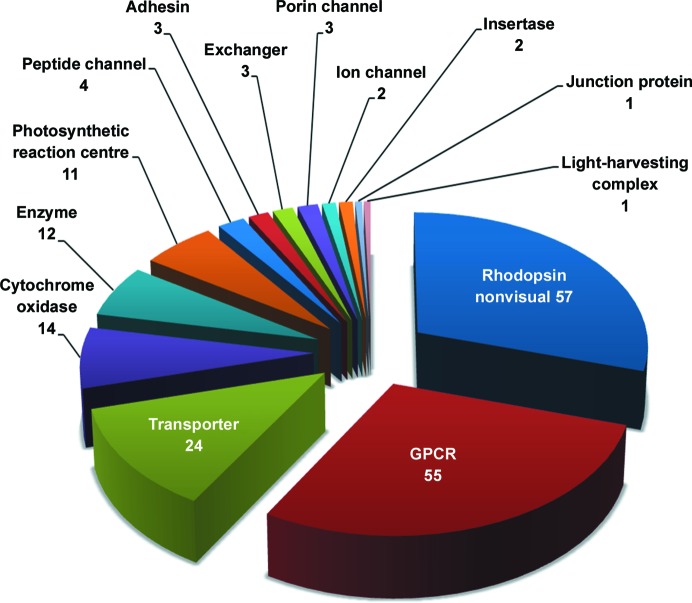
Distribution by biological function or activity of integral membrane proteins and peptides crystallized by the *in meso* method that have yielded crystal structures and records in the Protein Data Bank. The data correspond to the entries in Table 1 ▶ and were sourced from the Protein Data Bank in September 2014.

As of this writing, there are close to 200 records in the Protein Data Bank (PDB; Berman *et al.*, 2003 ▶; http://www.rcsb.org) attributable to the lipid cubic phase (LCP) or *in meso* method of crystallizing membrane proteins (Figs. 1 ▶ and 2 ▶, Table 1 ▶). The first appeared some two decades ago in 1996. Remarkably, almost half of the records have been deposited in the PDB since the beginning of 2012. This attests to the explosive growth in the rate at which the method is being used.

The *in meso* method has had some high-profile successes of late. These include the β_2_-adrenergic receptor–G_s_ protein complex, a structure that figured prominently in the 2012 Nobel Prize in Chemistry awarded to Robert Lefkowitz and Brian Kobilka (Rasmussen, Choi *et al.*, 2011 ▶), and channelrhodopsin, of opto­genetics fame (Kato *et al.*, 2012 ▶). Such notoriety undoubtedly contributes to interest in the method. However, a broader adoption of the method is more likely to reflect the success that it has had with an impressive range of membrane proteins and complexes. The equipment, materials and supplies needed to set up *in meso* crystallization are, for the most part, now available commercially, making the method more generally accessible. Also important are detailed protocols supported by open-access online instructional videos to aid the neophyte get up and running with little effort and at low cost. In the following, a comprehensive review is presented of the *in meso* method as applied to membrane proteins, including its recent application in the area of *in situ* serial crystallography. The use of the method with soluble proteins is also reviewed.

## 2. A working model for *in meso* crystallization   

A proposal has been advanced for how *in meso* crystallogenesis takes place at the molecular level (Fig. 3 ▶; Caffrey, 2008 ▶). It typically begins with an isolated biological membrane that is treated with detergent to solubilize the target protein. The protein–detergent complex, in the form of a mixed micelle, is purified by standard wet-laboratory biochemical methods. Homogenizing with a monoacylglycerol (MAG) effects a uniform reconstitution of the purified protein into the bilayer of the cubic phase. The latter is bicontinuous in the sense that both the aqueous and bilayer compartments are continuous in three dimensions. Upon reconstitution, the protein ideally retains its native conformation and activity and has complete mobility within the plane of the cubic phase bilayer. A precipitant is added to the mesophase, which triggers a local alteration in mesophase properties that include phase identity, microstructure, long-range order and phase separation. Under conditions leading to crystallization, one of the separated phases is enriched in protein, which supports nucleation and progression to a bulk crystal. The hypothesis envisions a local lamellar phase that acts as a medium in which nucleation and three-dimensional crystal growth occur. Molecular-dynamics simulations highlight the hydrophobic/hydrophilic mismatch between the protein and the surrounding bilayer in the lamellar phase as a driving force for oligomerization in the membrane plane (Khelashvili *et al.*, 2012 ▶; Johner *et al.*, 2014 ▶). The local lamellar phase also serves as a conduit or portal for proteins on their way from the cubic phase reservoir to the growing face of the crystal. Initially at least, the proteins leave the lamellar conduit and ratchet into the developing crystal to generate a layered (type I) packing of protein molecules. Given that proteins reconstitute across the bilayer of the cubic phase with no preferred orientation and the three-dimensional continuity of the mesophase, it is possible for the resulting crystals to be polar or nonpolar. These correspond to situations in which adjacent proteins in a layer have their long-axis director oriented in the same or in opposite directions.

The proposal for how nucleation and crystal growth come about *in meso* relies absolutely on the three-dimensional continuity of the mesophase. Under the assumption that the sample exists as a single, liquid crystallite or mono-domain, continuity ensures that the mesophase essentially acts as an infinite reservoir from which all protein molecules in the sample can end up in a bulk crystal. Neither the lamellar liquid crystal (L_α_) nor the inverted hexagonal (H_II_) phases, both of which are thermodynamically accessible mesophases in lipidic systems, have three-dimensional continuity and, alone, are unlikely to support membrane-protein crystallogenesis by the *in meso* method.

However, it is possible to envision crystal growth that occurs by way of a local H_II_ phase (Caffrey, 2011 ▶, 2013 ▶). Indeed, there are several crystallization conditions, such as high salt, that favour this mesophase and that support crystal growth. As is the case with the cubic and lamellar phases, the cubic and H_II_ phases can and do co-exist (Caffrey, 1987 ▶). Transitions between the two require inter-mesophase continuity. Since bitopic and polytopic membrane proteins span the bilayer at least once, the need to remain integral to the bilayer also prevails in the H_II_ phase. Indeed, locations where this can happen exist throughout the H_II_ phase, specifically at points of closest contact between lipid-coated, water-filled rods. At such locations proteins can diffuse one-dimensionally along the length of the H_II_ phase rods to associate with one another – along and between rods – first in nuclei that, in time, evolve into macroscopic crystals. As with the lamellar phase model, the cubic phase will act as a reservoir to provide a continuous supply of proteins to the growing face of the crystal. A consequence of this growth-mechanism type is that crystal packing, initially at least, will be hexagonal as opposed to layered or type I.

That the *in meso* method works with bitopic and polytopic proteins, having one or several membrane crossings, respectively, has been well proven. It has also been shown to support the crystallization of water-soluble proteins (§[Sec sec16]16). A mechanism for how this comes about has been presented (Caffrey, 2008 ▶). While as yet there are no examples in the literature of the method working with monotopic or lipid-anchored proteins, we can anticipate these emerging in the not too distant future. A simple mechanism for crystallization of anchored proteins could involve a form of interdigitation. Here, the acyl chains of lipid monolayers with which the protein are associated interpenetrate across the bilayer mid-plane. This would enable contact between proteins in and orthogonal to the membrane plane, facilitating three-dimensional nucleation and crystal growth. For monotopic proteins with membrane-integral domains that can contact across the bilayer mid-plane, three-dimensional nucleation and crystal growth can come about as described above for bitopic and polytopic proteins. With peripheral or ‘weakly’ monotopic targets, and indeed for lipid-anchored proteins, a variation of the mechanism of crystallization envisioned for soluble proteins (§[Sec sec16]16) could be invoked.

Because of the proposed need for the diffusion of proteins in the bilayer and of precipitant components in the aqueous channels of the mesophase, the expectation is that crystal-growth rates might be tardy *in meso*. However, crystals have been seen to form within an hour, which suggests that the slowness associated with restricted diffusion can be compensated for by a reduction in dimensionality (Caffrey, 2003 ▶, 2008 ▶). The latter is a result of the protein being confined to a lipid bilayer with its long axis oriented perpendicular to the membrane plane. Thus, the number of orientations that must be sampled to effect nucleation and crystal growth is few *in meso* compared with its *in surfo* counterpart that employs surfactant micelles and in which all of three-dimensional space is accessible.

That crystal growth takes place in a mesophase implies that it is happening in a convection-free environment. This is analogous to growth under conditions of microgravity or in a gel or a micro-fluidic channel, which offer the advantage of a stable zone of depletion around the growing crystal and thus slower and more orderly growth (Caffrey, 2000 ▶). Settling of crystals and subsequent growth into one another are also avoided under these conditions, as is the likelihood that impurities are wafted in from the surrounding solution to poison the face of the crystal and limit growth. For all these reasons *in meso* crystallogenesis is similar to crystallization in space, with the prospect of producing high-quality, structure-grade crystals.

## 3. The *in meso* method: practical issues and challenges   

Setting up an *in meso* crystallization trial is straightforward (Fig. 4 ▶). Typically, it involves combining two parts protein solution with three parts lipid at 20°C (Caffrey & Cherezov, 2009 ▶; Caffrey & Porter, 2010 ▶). The most commonly used lipid is the monoacylglycerol (MAG) monoolein. According to the monoolein–water temperature–composition phase diagram (Fig. 5 ▶; Qiu & Caffrey, 2000 ▶), and assuming there is no major influence on the phase behaviour of the protein-solution components, this mixing process should generate, by spontaneous self-assembly, the cubic mesophase at or close to full hydration. The original method for mixing lipid and protein solution involved multiple, cumbersome centrifugations in small glass tubes. Harvesting crystals required cutting the tubes and searching for small crystals through curved glass, which was not easy, very inefficient and required experience, time and patience.

The cubic phase is sticky and viscous in the manner of thick toothpaste (Fig. 6 ▶). As such, it is not easy to handle. In the course of earlier lipid-phase science work carried out in the Membrane Structural and Functional Biology (MS&FB) group, we had developed tools and procedures for manipulating such refractory materials. One of these, the coupled-syringe mixing device (Fig. 4 ▶; Cheng *et al.*, 1998 ▶), was ideally suited to the task of combining microlitre volumes of monoolein with membrane-protein solution in a way that produces protein-laden mesophase for direct use in crystallization trials with minimal waste. The mixer consists of two, positive-displacement Hamilton micro-syringes connected by a narrow-bore coupler. Lipid is placed in one syringe and protein solution in the other. Mixing is achieved by repeatedly moving the contents of the two syringes back and forth through the coupler (Caffrey & Porter, 2010 ▶). The coupler is replaced by a needle for convenient dispensing of the homogenous mesophase into wells of custom-designed, glass sandwich crystallization plates (Cherezov & Caffrey, 2003 ▶; Cherezov *et al.*, 2004 ▶). Precipitant solutions of varying compositions are placed over the mesophase and the wells are sealed with a cover glass. For initial screening, the plates are incubated at 20°C and monitored for crystal growth. The optical quality is the best it can be given that the mesophase is held between two glass plates and the mesophase itself is transparent (Fig. 7 ▶). This means that crystals of just a few micrometres in size can readily be seen by microscope whether the proteins are coloured or not. The use of cross-polarizers can enhance the visibility of small crystals, which usually appear birefringent on a dark background; the cubic phase itself is optically isotropic and non-birefringent. An added feature of the glass sandwich plates is that the double-sided tape used to create the wells provides almost hermetic sealing, ensuring minimal change in well composition during the course of trials that can last for months. Step-by-step instructions, complete with an open-access online video demonstration of the entire *in meso* crystallization process, have been published (Caffrey & Cherezov, 2009 ▶; Caffrey & Porter, 2010 ▶; Li, Boland, Aragão *et al.*, 2012 ▶; Li, Boland, Walsh *et al.*, 2012 ▶).

## 4. High-throughput crystallogenesis and the *in meso* robot   

The protocol just described refers to the manual mode of setting up crystallization trials. Accurate and precise delivery of the protein-laden mesophase in volumes that range from picolitres to microlitres was made possible by the use of an inexpensive repeat dispenser in combination with differently sized micro-syringes (Fig. 4 ▶ *e*; Cherezov & Caffrey, 2005 ▶, 2006 ▶; Caffrey, Eifert, 2014 ▶). The smaller volumes mean that the *in meso* method works with miniscule quantities of target protein. Thus, extensive crystallization trials can be set up with just a few micrograms of valuable membrane protein, making the *in meso* method one of the most efficient in terms of protein (and lipid and ligands, as appropriate) requirement.

Whilst the repeat dispenser greatly facilitated the *in meso* method, it was still a manual setup with limits to the number of trials that one person could comfortably and reproducibly set up at a sitting. The need to automate the process was obvious. With the assistance of A. Peddi and Y. Zheng, engineers at The Ohio State University where the original work was carried out, we were able to perform a proof-of-principle robotics exercise employing *LabView*-controlled motorized translation stages operating and supporting a micro-syringe and a crystallization plate. The prototype was used to demonstrate that the viscous mesophase could be dispensed automatically and wells filled in such a way that eventually yielded crystals. This was sufficient to secure funding for a robot, which was custom-designed and built to our specifications (Cherezov *et al.*, 2004 ▶).

The *in meso* robot has two arms programmed to move simultaneously. One dispenses the viscous protein-laden mesophase, while the other dispenses precipitant. Typical volumes used are 30–50 nl mesophase (consisting of 12–20 nl protein solution and 18–30 nl monoolein) and 600–800 nl precipitant solution. Custom 96-well glass sandwich plates were designed which take several minutes to fill using an eight-tip robot. The robot enables the precise and accurate setting up of *in meso* crystallization trials with picolitre to microlitre volumes of mesophase in high-throughput mode and, if required, under challenging conditions of reduced temperature and controlled lighting. Given the success of the *in meso* robot, several are currently in use in laboratories throughout the world. Variations on the original design, in which tip alignment is performed automatically and in which precipitant is handled by disposable tips, are now commercially available. Another uses a 96-tip liquid-dispensing head to deliver precipitant solution in a single action, thereby reducing the time taken to set up a single plate to less than 2 min. These and other commercially available robots represent important advances that simplify the *in meso* setup and make the method more generally available and user-friendly.

With the success that the *in meso* method has had, it is perhaps not unexpected to find products appearing on the market in support of this now proven, robust crystallogenesis approach. In addition to the *in meso* robots, these include a number of precipitant screen kits, glass and plastic sandwich plates, and a plate that comes complete with lipid-coated wells. The vendors indicate that the latter can be used with a liquid-dispensing robot for protein-solution delivery first and precipitant post-swelling.

## 5. Mesophase compatibility with protein-solution components   

As alluded to above, what happens during *in meso* crystallization is intimately tied up with mesophase behaviour (Fig. 3 ▶; Caffrey, 2008 ▶). The working hypothesis for how nucleation comes about begins with the protein reconstituting into the continuous bilayer of the cubic phase. Precipitant is added, which triggers the local formation of a lamellar phase into which the protein preferentially partitions and concentrates in a process that leads to nucleation and crystal growth. Experimental evidence in support of aspects of this model has been reported (Cherezov & Caffrey, 2007 ▶; Caffrey, 2008 ▶).

Experience built up over several years of working with the *in meso* method suggests that the mesophase behaviour observed during the course of crystallization mimics that of the monoolein–water system (Fig. 5 ▶). The implication therefore is that the protein solution has little effect on the behaviour of the hosting lipid mesophase into which the protein is reconstituted. This solution, along with the target protein, typically includes lipid, detergent, buffers and salt at a minimum. Other components, such as glycerol, sulfhydryl reagents, denaturants *etc.*, are not uncommon. Each of these can impact on phase behaviour and, by extension, the outcome of a crystallization trial. In the interests of learning about component compatibility, the sensitivity of the monoolein–water cubic phase system to their inclusion has been evaluated. Our findings indicate that the default cubic mesophase is remarkably resilient and retains its phase identity and microstructure in the presence of a vast array of different additives. These include glycerolipids, cholesterol, free fatty acids, detergents, denaturants, glycerol and sulfhydryl reagents, among others (Ai & Caffrey, 2000 ▶; Cherezov *et al.*, 2001 ▶, 2002 ▶; Misquitta & Caffrey, 2003 ▶; Clogston & Caffrey, 2005 ▶; Clogston *et al.*, 2005 ▶; Liu & Caffrey, 2005 ▶, 2006 ▶; Cherezov, Clogston *et al.*, 2006 ▶; Cherezov, Yamashita *et al.*, 2006 ▶). Of course, for each there is a concentration beyond which the cubic phase is no longer stable. In most cases, these limits have been identified.

Occasionally, the concentration of a protein-solution component is not known exactly. Detergent is a case in point. This poses a problem because if there is too much detergent the bulk lamellar phase may form but will not support crystallization (Ai & Caffrey, 2000 ▶; Misquitta & Caffrey, 2003 ▶). It may also be that a new detergent is being used whose compatibility with the cubic phase is not known. In this case, a small amount of the buffer employed to solubilize the protein or, preferably, the protein solution itself can be used to prepare the mesophase. The physical texture, appearance between crossed polarizers and/or small-angle X-ray scattering (SAXS) behaviour of the mesophase will indicate which phase has been accessed. If, for example, it is a lamellar phase that forms, suggesting too much detergent, then another purification step in which its concentration in the final protein solution is reduced may be sufficient to solve the problem. We have encountered situations with bacteriorhodopsin where the particular preparation ended up having an excess of detergent. The mesophase first formed was lamellar, but when it was used in combination with certain precipitants a transition back to the cubic phase was induced and the sample went on to grow crystals (Misquitta & Caffrey, 2003 ▶; Caffrey, 2008 ▶). This highlights the importance of understanding mesophase behaviour for more rational and productive crystallization.

## 6. Screen-solution compatibility   

As noted, *in meso* crystallization relies on a bicontinuous mesophase which acts as a reservoir to feed protein into nucleation sites and for crystal growth. Crystallization screening requires that chemical space be interrogated over wide limits to find conditions that support crystallogenesis. In the screening process, therefore, the protein-laden mesophase is exposed to precipitant solutions that encompass hundreds and perhaps thousands of different chemical compositions. Screen-solution components typically include buffers that cover a wide pH range, polymers, salts, small organics, detergents, apolar solvents, amphiphiles *etc.*, and all at different concentrations. Each component can potentially destabilize the mesophase. In a separate study using SAXS, we examined the compatibility of the default monoolein–water cubic phase with various commonly used precipitant screen solutions (Cherezov *et al.*, 2001 ▶). What we found was hardly surprising. Compatibility was temperature-dependent and the usual suspects, which included organic solvents, destroyed the cubic phase, rendering these screen solutions effectively useless. A goal of the study was to design screens that were mesophase-friendly. However, this goal was never pursued; instead, we have opted for the convenience of commercial screen kits mindful of the fact that certain conditions are not useful. As a result, certain kits are simply not used because they contain too few conditions that are compatible with the cubic phase.

## 7. Sponge phase   

During the course of mesophase compatibility studies, we noticed that particular screen components caused the cubic phase to ‘swell’ and, under certain conditions, to form what is referred to as the sponge phase. The latter evolves from the cubic phase as a result of the ‘spongifying’ component lowering the bilayer interfacial curvature and presumably its bending rigidity (Chung & Caffrey, 1994 ▶), thereby enabling the mesophase to absorb more lyotrope (aqueous solution). This is evident in the SAXS pattern, where the lattice parameter of the cubic phase rises with spongifier concentration. Eventually, the mesophase loses order and the low-angle diffraction pattern becomes diffuse. Fortunately, the sponge phase retains its bicontinuity and, as a result, can support *in meso* crystallogenesis (Cherezov, Clogston *et al.*, 2006 ▶; Caffrey, 2008 ▶; Wöhri *et al.*, 2008 ▶). One advantage of the sponge phase is that its aqueous channels are dilated. Thus, proteins with large extramembrane domains should be accommodated in and amenable to crystallogenesis from the sponge phase (§[Sec sec8.2]8.2). Further, the reduced interfacial curvature and bending rigidity are likely to facilitate more rapid and long-range diffusion within the lipid bilayer. Since net movement of protein from the bulk mesophase reservoir to nucleation and growth sites is a requirement for crystallization, this effect alone should contribute to improved crystallization, provided, of course, that the process is not too fast. Interestingly, many of the proteins that have yielded to the *in meso* method have been crystallized under conditions that favour sponge-phase formation (Table 1 ▶; Caffrey *et al.*, 2012 ▶).

Reflecting the utility of the sponge phase for *in meso* crystallo­genesis, a number of commercial screening kits now include spongifiers such as polyethylene glycol (PEG), Jeffamine and butanediol, among others. Some of these provide a preformed sponge phase to which the protein solution is added directly. We continue to use the original method that involves an active protein-reconstitution step and where the entire crystallization screen space is available for sampling.

## 8. Rational host lipid design   

### 8.1. Low-temperature crystallogenesis   

The MS&FB group has devoted considerable time and effort to establishing rules for rationally designing lipids with specific end uses. One such application concerned the development of a host lipid for use in *in meso* crystallogenesis at low temperatures. Certain proteins are labile and require handling at low temperatures. A potential problem with the *in meso* method, in the default mode at least, is that it relies upon monoolein as the hosting lipid. The cubic phase formed by monoolein is not thermodynamically stable below about 17°C (Qiu & Caffrey, 2000 ▶) and performing crystallization trials in a cold room at 4–6°C is risky. For this low-temperature application, therefore, a *cis*-monounsaturated monoacylglycerol, 7.9 MAG, was designed using the rules referred to above. The target MAG was synthesized and purified in-house and its phase behaviour was mapped out using SAXS (Misquitta, Cherezov *et al.*, 2004 ▶). As designed, it produced a cubic phase stable in the range from 6 to 85°C, as designed. 7.9 MAG has been used in the crystallization of a number of membrane proteins in the MS&FB group and beyond. It, along with other synthetic and natural MAGs (see below), are available to the community by way of commercial vendors.

The word ‘risky’ was used in the previous paragraph in reference to low-temperature crystallization with monoolein as the hosting lipid. This reflects the fact that it is possible to perform successful *in meso* work with monoolein at 4°C provided that the system undercools. Fortunately, the cubic phase is noted for this capacity (Fig. 5 ▶; Briggs & Caffrey, 1994 ▶; Qiu & Caffrey, 2000 ▶), and we regularly perform successful crystallization trials with monoolein in the 4–17°C range. As expected, under these metastable conditions the mesophase will occasionally convert to the lamellar crystalline or solid phase, which is useless as far as crystallization is concerned.

Sugar-phytane lipids have been synthesized that form the fully hydrated cubic phase in the 10–70°C range (Hato *et al.*, 2004 ▶) and that might find application for *in meso* crystallization at reduced temperatures.

### 8.2. Proteins and complexes with large membrane footprints and large extramembrane domains   

In the following, two recent examples of lipids rationally designed for use in crystallizing targets with large footprints in the plane of the membrane and/or extensive extramembrane domains are described. The first refers to cytochrome *caa*_3_ oxidase from *Thermus thermophilus* (Lyons *et al.*, 2012 ▶). This terminal oxidase is a large 120 kDa heterotrimeric protein with 23 transmembrane helices and a cytochrome *c*-like extramembrane domain as a C-terminal extension to one of its subunits. Extensive crystallization trials using traditional vapour-diffusion methods failed to produce structure-grade crystals. The *in meso* method was considered as an appropriate alternative. At the time that the study was undertaken, *in meso* crystallization had generated crystals and a structure of light-harvesting complex II, LH_II_, whose bulk in the plane of the membrane resembled that expected for *caa*_3_. Initial *in meso* trials with the default lipid, 9.9 MAG or monoolein, failed to produce useful crystals. Anticipating the likelihood that 9.9 MAG would not suit every membrane protein, the lipid-synthesis program in the MS&FB Group (Caffrey *et al.*, 2009 ▶; Yang *et al.*, 2012 ▶) provided alternative MAGs with which to screen for crystallogenesis. The first of these tested was 7.7 MAG, which has an acyl chain 14 carbon atoms long and a *cis* double bond between carbon atoms 7 and 8. 7.7 MAG had been shown to form a cubic mesophase with a thinner, less highly curved bilayer and with enlarged aqueous channels (Misquitta, Misquitta *et al.*, 2004 ▶). A thinner bilayer was considered to be desirable for use with *caa*_3_ because it more suitably complemented the hydrophobic thickness predicted for related cytochrome oxidases of known structure. Additionally, the larger aqueous channels provided by 7.7 MAG were attractive in the context of *caa*_3_ with its added extramembrane bulk in the form of a cupredoxin and the aforementioned tethered cytochrome-*c*-like domain. As expected, 7.7 MAG produced crystals; upon optimization they provided a structure at 2.36 Å resolution (Lyons *et al.*, 2012 ▶).

The second example is the β_2_-adrenergic receptor–G_s_ protein complex (Rasmussen, DeVree *et al.*, 2011 ▶). Earlier work had shown that the receptor alone produced structures to high resolution with the default lipid 9.9 MAG using the *in meso* method. However, efforts to grow structure-grade crystals of the receptor as a complex with its cognate G_s_ protein in monoolein failed. The G_s_ protein is itself a large heterotrimeric complex with a molecular weight of ∼80 kDa. It binds to the exposed intracellular surface of the receptor and adds considerable extramembrane bulk to the target. In this particular instance the G_s_ protein had bound to it a camelid single-chain antibody or nanobody (15 kDa), and T4 lysozyme (19 kDa) was fused to the N-terminus of the receptor. Both contributed additional extramembrane heft to the complex. Given that the cubic phase prepared with monoolein alone has aqueous channels in which the water-soluble domains must reside that are only 50 Å in diameter, failure to crystallize in monoolein did not come as a surprise. 7.7 MAG, with its significantly larger aqueous channels, was immediately identified as a suitable alternative host lipid and, with some limited optimization, it generated diffraction-quality crystals and a structure of the complex (Rasmussen, DeVree *et al.*, 2011 ▶). Interestingly, the precipitant used for the production of the final crystals included PEG 400, a known spongifier, and the crystals were harvested from what appeared to be a sponge phase. It seems likely therefore that the short-chain MAG and the spongifier worked hand in hand to generate a bicontinuous medium that accommodated unrestricted diffusion and that facilitated crystallization of the complex with its extensive extramembrane domains. Future *in meso* crystallization trials with targets of this sort will undoubtedly benefit from the use of alternative MAGs in concert with sponge phase-inducing precipitants. Commercial crystallization kits that include such materials are likely to be forthcoming. It is important to note that in all of the aforementioned GPCR work the host MAG was doped with cholesterol (see the following section).

## 9. Lipid screening   

### 9.1. Additive lipid   

Early on in the development of the *in meso* method, the author recognized that monoolein, as the lipid used to create the hosting mesophase, is a most uncommon membrane lipid. The sense was that this lipid might rightly be regarded as foreign by certain target proteins and cause them to destabilize or to adopt an unnatural conformation. One possible solution was to use a native membrane lipid that would form the requisite cubic phase at 20°C. None were available. An alternative was to use monoolein, or another suitable MAG, as the hosting lipid and to augment it with typical membrane lipids, thereby creating a more native-like environment. Accordingly, the carrying capacity of the monoolein cubic phase for a number of different lipids was established using SAXS (Cherezov *et al.*, 2002 ▶). This capacity amounted to about 20 mol% in the cases of phosphatidylcholine, phosphadidylethanolamine and cholesterol, with lesser amounts of phosphatidylserine and cardiolipin being accommodated. The approach of using additive lipids has had spectacular successes in the GPCR field, where cholesterol doping of the cubic phase was and continues to be critical to the production of structure-grade crystals (Caffrey *et al.*, 2012 ▶). Recently, it proved to be crucial in obtaining a high-resolution structure of human microsomal prostaglandin E2 synthase 1 (mPGES1), where the host lipid, 8.8 MAG, was doped with 2 mol% DOPC (Li *et al.*, 2014 ▶).

### 9.2. Host lipid   

Monoolein was the first lipid used for *in meso* crystallogenesis. From the outset, it was recognized that this one lipid may not work with all target membrane proteins (Caffrey, 2003 ▶; Misquitta, Cherezov *et al.*, 2004 ▶). These, in turn, come from a variety of native membranes which differ in lipid composition, surface charge and packing density, fluidity and polarity profile, bilayer thickness, intrinsic curvature, bending elasticity, *etc.* Thus, having a range of MAGs that differ in acyl-chain characteristics with which to screen was deemed to be important. Using principles of rational design, several MAGs were identified with the requirement that they form the cubic phase at 20°C under conditions of full hydration. A number of lipids meeting this specification have been synthesized and characterized in-house. They constitute a very successful host-lipid screen in the MS&FB group and beyond. With several targets, which include β-barrels, α-helical proteins, a GPCR–G_s_ protein complex and an integral peptide antibiotic, crystals have been grown by the *in meso* method using these alternative MAGs (Misquitta, Cherezov *et al.*, 2004 ▶; Misquitta, Misquitta *et al.*, 2004 ▶; Höfer, Aragão, Lyons *et al.*, 2011 ▶; Li *et al.*, 2011 ▶, 2015 ▶; Li, Shah *et al.*, 2013 ▶; Ring *et al.*, 2013 ▶; Lyons *et al.*, 2014 ▶; Malinauskaite *et al.*, 2014 ▶; Takeda *et al.*, 2014 ▶). In a number of cases monoolein either failed to produce crystals or the crystals that it did produce were not of diffraction quality. It was only when MAGs from the host-lipid screen were used that structure-grade crystals were obtained. A number of these novel MAGs are available to the community through commercial suppliers.

## 10. When protein concentration is low   

The driving force for nucleation is greater the more supersaturated the system is. Thus, a common strategy in the area of crystallization is to work at the highest possible protein concentration to favour nucleation, and to lower the protein concentration subsequently to just above the (super)solubility limit for the slow, orderly growth of a few, good-quality crystals. It is likely that the same principles apply to crystallization *in meso*, where initially the highest possible protein concentration should be used in support of nucleation. There are at least two issues that must be dealt with in this context that apply to membrane proteins. Firstly, most membrane proteins are prepared and purified in combination with detergents. Thus, the detergent is carried along with the protein into the crystallization mixture. It follows then that as the protein concentration increases, the detergent concentration will rise in parallel. This may work against crystallization because high levels of detergent can destabilize the host mesophase (§[Sec sec5]5; Ai & Caffrey, 2000 ▶; Misquitta & Caffrey, 2003 ▶; Caffrey, 2008 ▶). Of course, the sensitivity to added detergent will depend, among other things, on the identities of the host lipid and detergent. Completely removing the detergent before folding the protein into the crystallization mixture is usually not an option because it is commonly required to keep the protein soluble as a mixed micelle. One alternative is to reduce the detergent load to an acceptable level before combining the protein with the host lipid. This can be performed with BioBeads or by eluting the protein in a highly concentrated form from an affinity column. Using detergents with low critical micelle concentration values, such as lauryl maltose neopentyl glycol (MNG-DDM), is also worth investigating.

Raising the protein concentration in the solution used to make the mesophase without simultaneously elevating the detergent concentration is an important goal to work towards. This can be approached by selecting only the peak fractions from a final polishing gel-filtration column and using the largest workable molecular-weight cutoff filters for protein concentration. Glycerol and urea can raise protein solubility and both are compatible with the cubic phase (§[Sec sec5]5; Li & Caffrey, 2014 ▶). If this approach is used, however, the additive should be removed or its concentration dramatically reduced prior to running *in meso* crystallization trials. Simply equilibrating the mesophase thus formed with excess precipitant under standard crystallization conditions (50 nl mesophase + 800 nl precipitant) will eventually reduce the additive concentration by 40-fold. Further reductions are possible following the protocol described in the next paragraph.

The second issue has to do with raising the concentration of protein in the lipid bilayer of the cubic phase to facilitate nucleation. Two approaches can be tried that are quite different but that achieve the same end. The first exploits the water-carrying capacity of the cubic phase, a property that varies with lipid identity (Misquitta, Misquitta *et al.*, 2004 ▶; Caffrey, 2008 ▶). Thus, the reconstituted protein will be more concentrated in the bilayer of a cubic phase prepared with a lipid of high water-carrying capacity than would be obtained for a less hydrating lipid. The second approach, referred to as the ‘cubicon’ method, involves sequential reconstitutions in which the protein concentration in the bilayer rises in each round (Li & Caffrey, 2014 ▶). The membrane protein preferentially partitions from the aqueous solution into the bilayer of the cubic phase. If the reconstitution step is repeated using a single mesophase bolus and with a series of solutions of protein at low concentration, the protein load in the bilayer of the mesophase will increase in each reconstitution round, leaving excess aqueous solution depleted of protein. This protein-depleted solution is removed before the next round of reconstitution commences. Successful applications of the cubicon approach have been implemented in the author’s laboratory for several integral membrane-protein targets (Li & Caffrey, 2014 ▶; P. Ma & M. Caffrey, unpublished work).

## 11. Cell-free expressed protein   

For the most part, *in meso* crystallization trials are conducted with naturally abundant proteins or proteins produced recombinantly in expression systems such as *Escherichia coli*, insect or mammalian cells. Cell-free expression is a method with considerable promise in the membrane-protein field (Schwarz *et al.*, 2007 ▶). It is easy to perform, milligram quantities of protein can be produced overnight and costs are reasonable. Because the system is open, labelling (with selenomethionine, for example) is simple, harvesting protein is straightforward and the newly synthesized protein is already relatively pure. The cell-free method has been used to express integral membrane proteins for structure determination. To date, however, only three have been crystallized *in surfo*, two of which have yielded crystal structures (Chen *et al.*, 2007 ▶; Wada *et al.*, 2011 ▶). These include the transporter EmrE at 3.8 Å resolution and a light-driven pump at 3.2 Å resolution. Therefore, while the method is proven, and indeed kits for *in vitro* expression are available commercially, it cannot be considered to be routine. Intrigued by what the cell-free method had to offer with regard to quality protein for structure work, we evaluated its applicability using the *in meso* crystallogenesis method with diacylglycerol kinase (DgkA) as a test protein. It worked spectacularly well. Milligram quantities of the kinase were produced overnight as aggregated protein, the protein was solubilized, reconstituted into the cubic phase and crystallized. Satisfyingly, the structure, solved to 2.3 Å resolution with little optimization of crystallization conditions, was remarkably similar to that of *in vivo*-produced protein (Boland *et al.*, 2014 ▶).

In the DgkA study just described, we chose to carry out expression in the absence of a membrane mimetic in part because the aggregated protein thus formed *in vitro* resembled the inclusion bodies that the protein overexpressed *in vivo* forms naturally and that had been used successfully for crystallography. However, it is possible to perform cell-free expression in the presence of a membrane mimetic for the direct production of functional protein. To date, detergent micelles, liposomes, nanodiscs and bicelles have been used for this purpose, and each has its own pros and cons. A logical extension to this approach is to use the bicontinuous lipid mesophase as an alternative receiving membrane mimetic with several attractive features. To begin with, the cubic phase comprises an essentially limitless reservoir for the expressed membrane protein throughout which it can diffuse. Secondly, the mesophase includes a familiar bilayered membrane in which the newly synthesized protein is likely to feel at home and to retain a native, functional fold. Thirdly, the bicontinuous nature of the mesophase means that both sides of the membrane-embedded protein are accessible, which is important for functional characterization and assay development. Fourthly, should certain proteins prove refractory to unaided insertion into the mesophase, translocon proteins can be added to facilitate the process. Fifthly, because of its sticky and viscous nature the mesophase is readily harvested for subsequent use as a system with which to perform biochemical, pharmacological and biophysical characterization. Finally, another consequence of the unique rheological properties of the mesophase is that it lends itself to miniaturization and to microarray-type applications for high-throughput screening. One of our immediate objectives is to use the protein-laden mesophase for direct *in meso* crystallization. Thus, by performing cell-free expression and *in meso* crystallization in tandem, the need to separately isolate and purify the protein is avoided, rendering the process from gene to crystal highly efficient in terms of time and cost whilst eliminating the potential damaging effects of solubilizing detergents.

## 12. Experimental phasing   

The structures solved using *in meso*-grown crystals have relied predominantly on molecular replacement for phasing. The challenges associated with experimental phasing derive, in part, from a low anomalous signal-to-noise ratio owing to a combination of background low-angle and wide-angle scatter from adhering mesophase and the need to work with small and sometimes poorly diffracting, radiation-sensitive crystals. As often as not, data must be collected in angular wedges from different parts of a single crystal or from multiple crystals, and merging data satisfactorily can be a challenge. Despite the difficulties, successes with experimental phasing have been reported. In the past three years alone, the following structures have been solved by this method: channelrhodopsin from *Chlamydomonas reinhardtii* (PDB entry 3ug9; mercury-MAD; Kato *et al.*, 2012 ▶); the Na^+^/Ca^2+^ exchanger from *Methanococcus jannaschii* (PDB entry 3v5u; samarium-SAD; Liao *et al.*, 2012 ▶); β-barrels from *E. coli* (PDB entry 4e1s; selenomethionine-SAD; Fairman *et al.*, 2012 ▶) and *Y. pseudotuberculosis* (PDB entry 4e1t; selenomethionine-SAD; Fairman *et al.*, 2012 ▶); DgkA from *E. coli* (PDB entry 3ze3; selenomethionine-SAD; Li, Lyons *et al.*, 2013 ▶); human mPGES1 (PDB entry 4bpm; sulfur-SAD; Li *et al.*, 2014 ▶); CDP-alcohol phosphotransferase (PDB entry 4o6m; selenomethionine-SAD; Sciara *et al.*, 2014 ▶); and claudin-15 (PDB entry 4p79; selenomethionine-MAD; Suzuki *et al.*, 2014 ▶). It would appear therefore that whilst challenging, experimental phasing is a reality and should not limit structure determination using crystals grown *in meso*. Indeed, with careful measurements, native sulfur-SAD phasing with a single *in meso* crystal is possible (Weinert *et al.*, 2014 ▶). A detailed case study of experimental phasing as applied to DgkA has recently been reported (Li *et al.*, 2015 ▶).

## 13. Activity assays *in meso*   

It is assumed that proteins reconstituted prior to crystallization are functional *in meso*. In the case of BtuB, this was examined by measuring substrate (cyanocobalamin; CNCbl) binding to the protein incorporated into the cubic phase (Cherezov, Yamashita *et al.*, 2006 ▶). Protein-free control samples exhibited no binding, whereas test *in meso* BtuB-containing samples displayed convincing visual evidence of substrate uptake (CNCbl is pink). Binding was shown by quenching of the intrinsic fluorescence of aromatic residues by CNCbl and by direct ligand binding to be tight, with an apparent *K*_d_ value of ∼1 n*M*. Similar *K*_d_ values have been reported for the native membrane-bound and micellarized form of the protein. Sialic acid binding to the adhesin OpcA, measured by fluorescence quenching as with BtuB, was identical *in meso* and in detergent solution (Cherezov *et al.*, 2008 ▶). Taken together, the data support the view that these β-barrel proteins reconstitute into the bilayer of the cubic phase in an active form prior to *in meso* crystallization.

Functional activity assays *in meso* have been extended to include membrane-protein enzymes (Li & Caffrey, 2011 ▶). In the case of DgkA, a coupled-enzyme assay was used. With phosphatidylglycerol phosphate synthase (PgsA), activity was quantified by direct assay. In both cases, the viscous, sticky and porous nature of the cubic phase was used to advantage in enabling spectrophotometric activity assays to be performed in a high-throughput multi-well microplate format. With both enzymes, the cubic mesophase served as a useful and a convenient nanoporous membrane mimetic that supported native-like activity.

Recent studies with the dopamine 2 long (D2L) and histamine 1 (H1) GPCRs indicate ligand binding in the nanomolar range based on radioligand assays (Darmanin *et al.*, 2012 ▶). In this study, the receptors were reconstituted into the cubic phase by a passive method and showed significantly enhanced specific binding compared with their detergent-solubilized counterparts.

## 14. *In meso* structures   

As of this writing, the *in meso* method accounts for almost 200 records in the PDB that relate to integral membrane proteins and peptides (http://www.pdb.org; Fig. 1 ▶, Table 1 ▶). This corresponds to about 10% of all published membrane-protein structures, representing a wide range of distinct membrane-protein types, sizes and oligomeric forms. With successes that include bacterial rhodopsins, light-harvesting complex II, photosynthetic reaction centres, β-barrels, GPCRs and a GPCR–G protein complex, transporters, channels, enzymes, cytochrome oxidases, channelrhodopsin, a membrane-protein insertase, tight-junction claudin-15 and an integral membrane peptide, the method has a convincing record of versatility and range. Each of these membrane-protein types represents larger families, the members of which become suitable candidates for *in meso* crystallogenesis. The GPCR family is a case in point, with almost 800 distinct GPCRs coded for in the human genome alone. Accordingly, the *in meso* method, in combination with the necessary protein-engineering and receptor-stabilization strategies, is now contributing to the generation of GPCR structures in what amounts to a production-line fashion. Evidence in support of this statement is the recent spate of receptor structures, almost 40 in the past two and a half years, courtesy of the *in meso* method. The same degree of success can be anticipated for other membrane-protein families. Transporters would appear to be moving in this direction (Fig. 1 ▶, Table 1 ▶).

The further development of the *in meso* crystallogenesis approach is an important goal for members of the MS&FB group. One direction this has taken concerns the utility of the method with small membrane proteins. A separate analysis performed using a model cubic phase under restricted conditions indicated that suitable targets would need to include at least five transmembrane helices (Grabe *et al.*, 2003 ▶). Our experience with the sponge-phase variant of the cubic phase suggested otherwise. Accordingly, the utility of the method with a ‘mini-protein’, the pentadecapeptide antibiotic linear gramicidin, was investigated. It worked remarkably well, providing a structure with a resolution of better than 1.1 Å (Höfer *et al.*, 2010 ▶; Höfer, Aragão & Caffrey, 2011 ▶; Höfer, Aragão, Lyons *et al.*, 2011 ▶). This result is significant because it highlights the utility of the method with proteins having small footprints in the plane of the membrane. which abound in nature and include a multitude of receptors and signalling proteins.

## 15. Serial crystallography   

### 15.1. With free-electron laser X-rays   

Serial femtosecond X-ray crystallography (SFX) is a relatively new method for collecting crystallographic information from small crystals fed into a free-electron laser (FEL) beam composed of high-fluence X-ray bunches mere femtoseconds long (Chapman *et al.*, 2011 ▶, 2014 ▶; Spence *et al.*, 2012 ▶). Each encounter between an X-ray bunch and a microcrystal (hit) ideally gives rise to a single, still diffraction pattern with greater than ten measurable reflections. Since the crystals are randomly oriented, collecting patterns from enough of them (typically many thousands) produces a complete data set of high redundancy for structure determination, to date by molecular replacement with just one exception (Barends *et al.*, 2014 ▶). Data are typically collected in an evacuated interaction sample chamber operated at 20°C. Despite the intensity of the X-ray bunch (∼10^12^ photons per bunch), each is of such short duration that insufficient time (the pristinity window) is available for the changes associated with radiation damage to progress sufficiently before the diffracted X-rays have departed (run) with their structural manifest to be recorded. This is referred to as ‘hit and run’ (Caffrey, Li *et al.*, 2014 ▶) or ‘diffraction before destruction’ SFX (Chapman *et al.*, 2014 ▶).

Until recently, a fluid medium had been used to ferry crystals of membrane proteins across the beam for SFX (Chapman *et al.*, 2011 ▶; Johansson *et al.*, 2012 ▶). The process involved voluminous flow rates. Because productive interactions between X-rays and crystals in the flowing stream were so infrequent, vast amounts of valuable membrane protein were required for data collection and most of the protein went to waste. Typically, only one in 25 000 crystals produced a useful diffraction pattern. Thus, for example, when photosystem I (PSI) crystals were used dispersed in a liquid jet, data collection required 10 mg of protein (Chapman *et al.*, 2011 ▶). By contrast, when photosynthetic reaction centre crystals were delivered dispersed in the more viscous lipid sponge phase, 3 mg of protein were needed (Johansson *et al.*, 2012 ▶). The idea was subsequently mooted that using the highly viscous LCP, in which the membrane-protein crystals can be grown by the *in meso* method, might provide a transport medium for SFX with better hit rates. As a result of being so viscous, the flow rate would be reduced dramatically. If high enough crystal densities in the LCP could be achieved, the rate of delivery of crystals and X-rays to the interaction region could be matched for a most efficacious use of both. The method is hereafter referred to as LCP-SFX.

LCP-SFX is appealing as a method because it offers the prospect of obviating some of the issues that arise with *in meso*-based structure determination using synchrotron X-radiation. With the *in meso* method, crystals are typically grown in a sealed glass sandwich plate. Harvesting crystals is a somewhat cumbersome process that can lead to substantial loss of crystals and to degradation in diffraction quality. Data collection at a synchrotron source is typically performed at 100 K. Such a frigid temperature can stabilize conformational substates, particularly in the side chains of the protein, that are not physiologically relevant and that are possibly misleading as far as functional interpretation is concerned (Fraser *et al.*, 2011 ▶). Radiation damage is also a major concern with synchrotron-radiation sources, where residues such as aspartate and glutamate are particularly prone to undergo decarboxylation (Burmeister, 2000 ▶). Damage can be mitigated to a degree with large crystals, beam attenuation and data collection at cryo-temperatures, often requiring many tens of crystals. In this context, LCP-SFX was attractive in that it offered what amounts to *in situ* data collection with micrometre- or nanometre-sized crystals at or close to the more physiologically relevant 20°C and the prospect of outrunning radiation damage.

A test of the proposed LCP-SFX idea was performed at the Coherent X-ray Imaging (CXI) instrument at the Linac Coherent Light Source (LCLS) over the course of seven 12 h data-collection shifts in March 2013. Diffraction data were collected on Cornell–SLAC Pixel Array Detector (CSPAD) detectors. Crystals were ported across the XFEL beam as a continuously extruded bolus of mesophase by means of a specially engineered LCP injector (Weierstall *et al.*, 2014 ▶). The feasibility study was spectacularly successful, yielding high-resolution structures for three integral membrane proteins that included diacylglycerol kinase (DgkA) and two GPCRs (Liu *et al.*, 2013 ▶, 2014 ▶; Caffrey, Li *et al.*, 2014 ▶); a fourth is in the works. Record low quantities of protein were needed to obtain structures. In the case of DgkA this amounted to just 220 µg protein and 42 µl cubic phase. Clearly, the method is on deck for use in novel ways with a host of other membrane proteins and complexes.

### 15.2. With synchrotron X-rays   

The LCP injector developed for SFX (§[Sec sec15.1]15.1; Weierstall *et al.*, 2014 ▶) mimics the gas-tight Hamilton micro-syringes used in the coupled-syringe mixing device and for dispensing mesophase into the wells of crystallization plates (Cheng *et al.*, 1998 ▶; Cherezov & Caffrey, 2005 ▶; §[Sec sec3]3). However, the LCP injector was designed to operate at much higher pressures and therefore can, in a leak-free manner, extrude the viscous, microcrystal-laden mesophase through a long, narrow-bore capillary for SFX. The problem with SFX measurements, however, is that XFEL facilities are in very short supply globally and are in great demand. It made sense therefore to look to other, more readily available bright X-ray sources, such as synchrotrons, with which to make use of the LCP injector. To date, successful tests of the injector have been carried out with crystals of lysozyme and of integral membrane proteins, and data of sufficient quantity and quality have been collected for structure determination (http://www.esrf.eu/home/news/general/content-news/general/novel-injector-allows-X-rays-to-map-membrane-proteins.html). Like SFX, this amounts to serial crystallography (SX) where data must be collected from thousands of micro-crystals jetted across the beam. The advantages of this approach include the fact that synchrotron beam time is more generally available and accessible, and that measurements are made on the naked mesophase, without attenuating and scattering windows, in air under close to *in situ* conditions, obviating the need for cumbersome, inefficient harvesting, and at the more physiologically relevant room temperature. However, there are challenges that include radiation damage, injector maintenance and operation by skilled personnel, and the need for a high density of micrometre-sized crystals in a mesophase that is dust-free to prevent injector clogging. It is early days in the development of this type of SX methodology. Time will tell whether it offers real advantages over other methods of data collection. If it does, it will take the pressure off XFEL sources, which can be used in applications for which they are uniquely suited.

## 16. Water-soluble proteins   

The *in meso* method was developed and is used primarily for crystallizing membrane proteins. However, it also works with soluble proteins. Lysozyme, insulin, α-lactalbumin and thaumatin are cases in point (Landau *et al.*, 1997 ▶; Cherezov *et al.*, 2004 ▶; Tanaka *et al.*, 2004 ▶; Aherne *et al.*, 2012 ▶). There may well be advantages to growing soluble protein crystals *in meso* that relate to the fact that it mimics crystallization in gels and under conditions of microgravity (§[Sec sec2]2; Caffrey, 2003 ▶). Such conditions stabilize the depletion zone and minimize the settling of crystals on top of one another and the wafting of contaminants to the growing surface of the crystal, all of which are associated with improved crystal quality.

A quick and easy protocol for crystallizing lysozyme by the *in meso* method, which gives 15–20 µm-sized crystals within an hour, has been developed (Aherne *et al.*, 2012 ▶). It is currently being used for instructional purposes and as a training tool. Increasingly, the approach is being used to test new applications of the cubic phase: for example, as a viscous, slow-‘flowing’ medium in which to port microcrystals of soluble proteins and complexes into an XFEL or synchrotron beam for efficient, high hit-rate SFX or SX (Caffrey, Li *et al.*, 2014 ▶; Liu *et al.*, 2014 ▶). Crystals can be grown *in situ* and used essentially as with membrane proteins. An alternative approach is to combine extant crystals with pre-formed mesophase to create a dispersion that can be loaded directly into the reservoir of the LCP injector for SFX or SX measurements. In the latter case, the mesophase would best be prepared with the mother liquor in which the soluble protein crystals grew. As with membrane proteins, MAGs with different acyl-chain characteristics and correspondingly different mesophase microstructures, transport properties and rheologies should prove to be useful for generating and porting crystals of the widest possible range of soluble protein targets.

## 17. An evolving *in meso* screening strategy   

As a community, we have close to two decades of experience with the *in meso* crystallization method. Have we learned any lessons that can be used for a more rational, less empirical approach to generating high-resolution structures with *in meso*-grown crystals? Certainly guidelines have emerged and several are presented below. Part of the problem with disseminating information of this type relates to the high-profile nature of many of the target proteins being reported. In consequence, much of the work is published in high-impact journals where space is at a premium and only the most essential experimental detail is included. The community suffers as a result by not being privy to the nature and extent of the screening performed that yielded the final structures. We have attempted to make good this deficit by reporting full details of the screening strategies implemented in the MS&FB Group with β-barrel and α-helical membrane-protein targets that have led to structures (Fig. 7 ▶; Li *et al.*, 2011 ▶, 2014 ▶, 2015 ▶; Li, Shah *et al.*, 2013 ▶). Some of the lessons learned from these assorted studies are summarized below in no particular order.

*Numbers*. Be prepared to set up and to screen a LARGE number of conditions. Fortunately, *in meso* crystallization is highly efficient and requires very small amounts of protein and lipid. For calibration purposes, the DgkA structure emerged on the basis of screening 4000 96-well plates over a period of three years (Li, Shah *et al.*, 2013 ▶; Li *et al.*, 2015 ▶). By contrast, about 300 plates set up in the space of a year provided the human mPGES1 structure (Li *et al.*, 2014 ▶). Even less time and effort was required to produce crystal structures of the apo and peptide-bound forms of POT transporters (Lyons *et al.*, 2014 ▶). The latter projects benefitted greatly from the lessons learned with DgkA.
*Temperature*. Perform trials initially at 20°C. At the very least, 4°C should be tested next. Because the cubic mesophase readily undercools (Qiu & Caffrey, 2000 ▶), 9.9 MAG, and in our experience several other MAGs, can be used in screens at 4°C.
*Host lipid*. Begin with 9.9 MAG unless you have prior knowledge that a different MAG is preferred. As needed, explore short-chain MAGs. A number are now available commercially. As of this writing, we would typically test 7.8, 7.7, 7.9, 9.7 and 8.8 MAG in that order.
*Additive lipid*. The choice of lipid is dictated by the target and prior knowledge in relation to its preferences regarding stability and function. To date cholesterol, DOPC and native phospholipids have been used successfully. Not only should the identity of the lipid be examined but also the concentration at which it is used. Further, the method of adding the lipid needs to be considered. With mPGES1 (Li *et al.*, 2014 ▶), adding it to the host lipid prior to reconstitution worked; adding it to the protein prior to reconstitution did not.
*pH*. Perform a wide pH screen as early in the process as possible. Try to avoid cacodylate, which contains the toxic and strongly X-ray absorbing and fluorescing heavy atom arsenic. If phosphate or other such buffers are used that are known to form insoluble salt crystals with cations such as calcium and magnesium, carefully check that the early-stage crystals indeed consist of protein. Fluoride salts often produce crystals *in meso*.
*Protein concentration*. This should be screened for early in the process. Use the highest protein concentration available and dilutions of the same. If detergent carry-over is excessive the higher protein concentrations tested could destabilize the cubic phase, as observed with mPGES1.
*Salts*. Perform a salt additive screen at final concentrations of 0.1 and 0.4 *M* (Li, Shah *et al.*, 2013 ▶; Li *et al.*, 2014 ▶). A second, and perhaps even a third, salt screen later in the screening/optimization process can prove invaluable in the identification of additional salt components that will progress the project towards a structure. A second salt screen proved crucial with mPGES1 (Li *et al.*, 2014 ▶).
*Additives*. Small diols, such as butanediol and hexanediol, help to drive the mesophase in the direction of the sponge phase. With mPGES1 (Li *et al.*, 2014 ▶), this increased the crystal size, especially when the mesophase was not in the sponge phase to begin with. In our experience, organic solvents such as alcohols and acetone, as found in the Hampton Research Additive Screen kit, are not useful for *in meso* crystallogenesis.
*Precipitants*. The precipitants used to date with the *in meso* method fall into two major categories. The first consists of polymers and polyols with the potential to spongify the lipid mesophase. Specific examples include PEG 400 (GPCRs), Jeffamine M600 (photosynthetic reaction centres), pentaerythritol propoxylate (light-harvesting complex II) and MPD (cobalamin transporter, BtuB, DgkA and mPGES1). The second employs a high concentration of salts. Examples include sodium/potassium phosphate for bacterio­rhodopsin and sodium acetate for AlgE. Given the diversity of the precipitants that have worked across all crystallization methods, it is still recommended that a broad initial screening be performed with commercial kits (Li *et al.*, 2011 ▶, 2014 ▶; Li, Shah *et al.*, 2013 ▶). As the database of *in meso*-based structures grows, particular types of screens will emerge for specific target types. A good example of this is the PEG 400-based screens that are proving to be highly successful with GPCRs and transporters.
*Ligands*. If the apo form of the target proves refractory to crystallogenesis, tight-binding low off-rate ligands, where available, can prove invaluable. This has been well proven with GPCRs, where every published *in meso* structure is of a liganded complex (Table 1 ▶; Caffrey *et al.*, 2012 ▶). Often these are added during protein expression and purification. If the ligand stabilizes the target (thermally), this is all the more reason for including it because stability and crystallizability would appear to be strongly positively correlated.
*Constructs*. Protein engineering can be hugely beneficial in the realisation of a crystal structure. DgkA (Li, Lyons *et al.*, 2013 ▶), mPGES1 (Li *et al.*, 2015 ▶) and channelrhodopsin (Kato *et al.*, 2012 ▶), and the entire set of GPCRs (Caffrey *et al.*, 2012 ▶), are cases in point. Engineering can be performed to stabilize the target, to provide crystal contacts, to prevent post-translational modification and to remove segments, disordered termini or loops, for example, that may interfere with crystallization. With all such modifications, it is important to evaluate the effect that the changes have on function. Small mono-domain, single-chain antibodies called nanobodies serve many similar roles to fusions and have proved successful in the GPCR field (Rasmussen, Choi *et al.*, 2011 ▶; Ring *et al.*, 2013 ▶).

## 18. Facts and figures online   

Further details regarding the structure and function of integral, anchored and peripheral membrane proteins are available online in a convenient and searchable database: the Membrane Protein Data Bank (MPDB; Raman *et al.*, 2006 ▶; http://www.mpdb.tcd.ie). Unfortunately, owing to a lack of resources, the database has not been updated since 2011. Whilst records in the MPDB include structure information directly from and hyperlinked to the PDB, they also contain additional useful data relating to the type of protein, the methods and materials used for structure determination and so on obtained from the source literature. Statistical analyses on the contents of the database, which, unlike the PDB, is limited to membrane proteins, can be performed and viewed conveniently online. Examples include ‘detergents used for membrane protein structure work’, ‘number of structures published annually by method’ and the like. Thus, while out of date, it still contains useful and searchable information. Perhaps, in time, it can be coupled to the PDB for automatic updating.

Over the years, the PDB has improved its search features. With more complete record annotation, hopefully to include full crystallization details, it may be that specialized databases such as the MPDB or that of Steve White’s group (http://blanco.biomol.uci.edu/mpstruc/) will become redundant. This is as it should be, given the resources available to the PDB. As of this writing (September 2014), a ‘Search’ of ‘Everything’ from the PDB homepage (http://www.pdb.org) under ‘lipidic cubic phase’ yields 113 records and all are relevant. (Interestingly, a search under ‘lipid cubic phase’ yields only 89 records.) However, by our reckoning, the PDB holds ∼192 such records (Figs. 1 ▶ and 2 ▶, Table 1 ▶). With this set of records, it is possible to create, at the press of the ‘Report’ button, a very useful ‘customizable table’ that can be sorted, filtered and eventually exported to an Excel file. It includes hyperlinks to PDB records and to most, but not all, annotated items in the standard PDB record. We look forward to enhanced functionality of this important, weekly updated resource.

## 19. Prospects   

**Figure 2:**
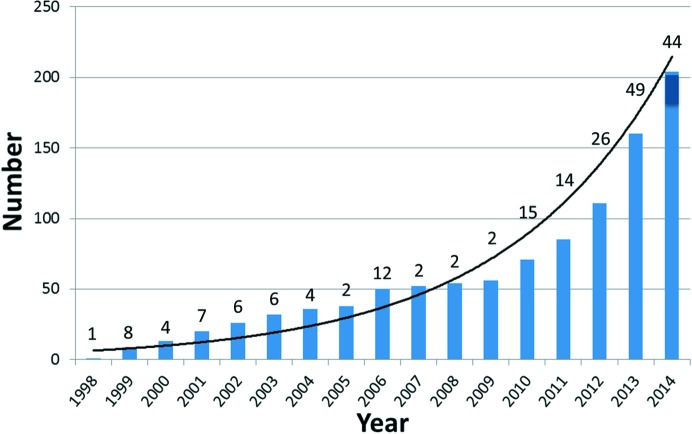
Annual cumulative number of released PDB records for integral membrane-protein and peptide structures solved with crystals grown by the *in meso* method. The number of records released each year is indicated. The figure for 2014 is estimated based on a count of 32 recorded up until September 2014. The line is drawn to guide the eye and takes the form *y* = 5.13exp(0.22*x*).

**Figure 3:**
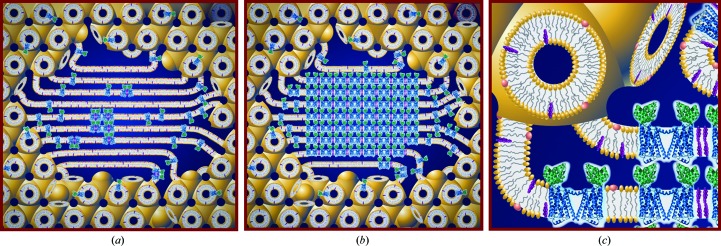
Cartoon representation of the events proposed to take place during the crystallization of an integral membrane protein from the lipid cubic mesophase. The process begins with the protein reconstituted into the curved bilayer of the ‘bicontinuous’ cubic phase (tan). Added ‘precipitants’ shift the equilibrium away from stability in the cubic membrane. This leads to phase separation, wherein protein molecules (*a*) diffuse from the bicontinuous bilayered reservoir of the cubic phase into a sheet-like or lamellar domain and (*b*) locally concentrate therein in a process that progresses to nucleation and crystal growth. Cocrystallization of the protein with native lipid (cholesterol) is shown in this illustration. As much as possible, the dimensions of the lipid (tan oval with tail), detergent (pink oval with tail), cholesterol (purple), protein (blue and green; β_2_-adrenergic receptor-T4 lysozyme fusion; PDB entry 2rh1), bilayer and aqueous channels (dark blue) have been drawn to scale. The lipid bilayer is ∼40 Å thick. An expanded view of the various components in the system is shown in (*c*). Reprinted from Li, Shah *et al.* (2013 ▶). Copyright 2013 American Chemical Society.

**Figure 4:**
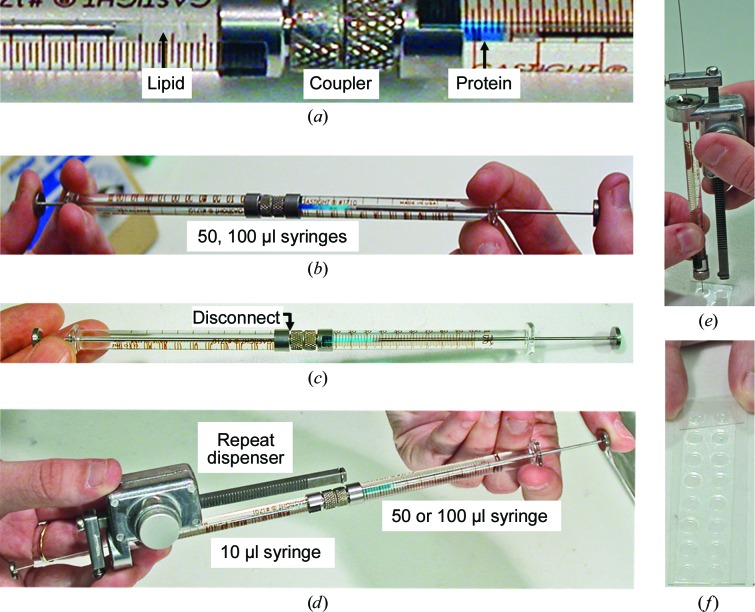
Setting up an *in meso* crystallization trial manually involves (*a*) placing membrane-protein solution and lipid into separate gas-tight micro-syringes (typically 50 or 100 µl) connected by a narrow-bore coupler, (*b*) passing the protein solution and lipid from one syringe to the other *via* the coupler to effect mixing, homogenization and spontaneous self-assembly of the cubic phase into the bilayer of which the protein has become reconstituted, (*c*) transferring the optically clear mesophase into one of the syringes, (*d*) replacing the empty syringe with a dispensing micro-syringe (typically 10 µl) mounted in a repeat dispenser and transferring protein-laden mesophase from the large to the small syringe by way of the coupler, (*e*) dispensing mesophase followed by precipitant solution into the wells of a glass sandwich crystallization plate and (*f*) sealing the wells with a glass cover slide. The remaining wells on the plate are filled and sealed and the plate is then incubated at the desired temperature to allow crystallization to occur. An open-access online video of the entire procedure is available (Caffrey & Porter, 2010 ▶).

**Figure 5:**
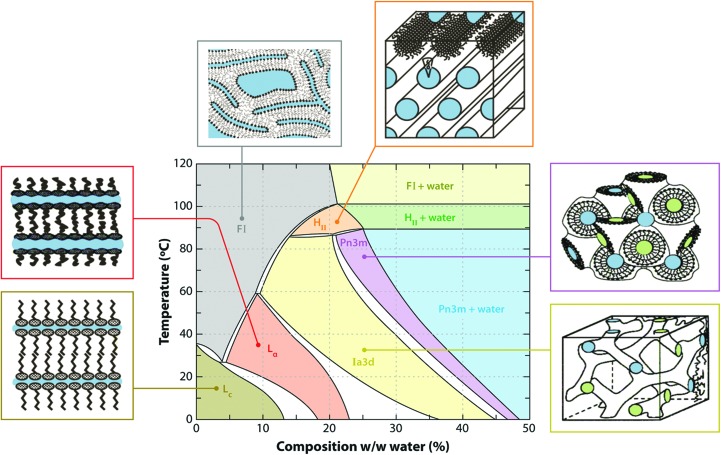
Temperature–composition phase diagram of the monoolein–water system determined under conditions of use in the heating and cooling directions from 20°C. A schematic representation of the various phase states is included, in which coloured zones represent water. The liquid crystalline phases below ∼17°C are undercooled and metastable (Qiu & Caffrey, 2000 ▶). Abbreviations: FI, fluid isotropic phase; H_II_, inverted hexagonal phase; L_α_, lamellar liquid crystalline phase; L_c_, lamellar crystal phase. Reprinted with permission from Caffrey (2009 ▶). Copyright (2009) Annual Reviews.

**Figure 6:**
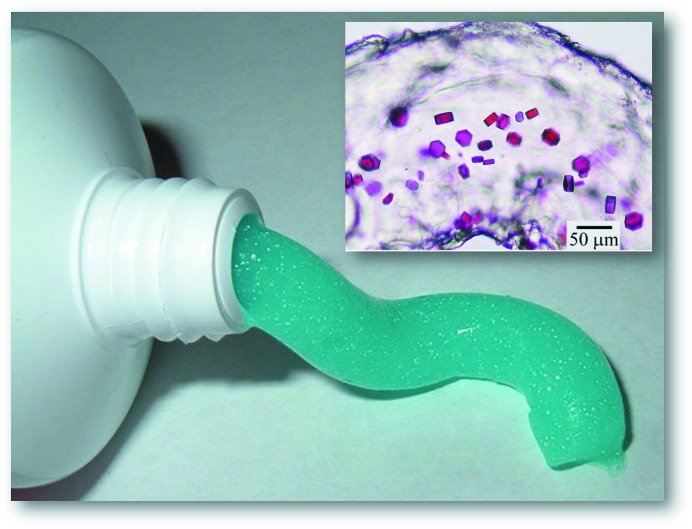
The cubic phase is viscous and sticky; it has the consistency of thick toothpaste. The particular toothpaste used in this figure contains small white crystals. This is exactly what is sought when *in meso* crystallization trials are set up. The inset shows crystals of the integral membrane light-driven proton pump bacteriorhodopsin growing in the lipid cubic phase. The analogy between the toothpaste and the crystal-laden mesophase is obvious.

**Figure 7:**
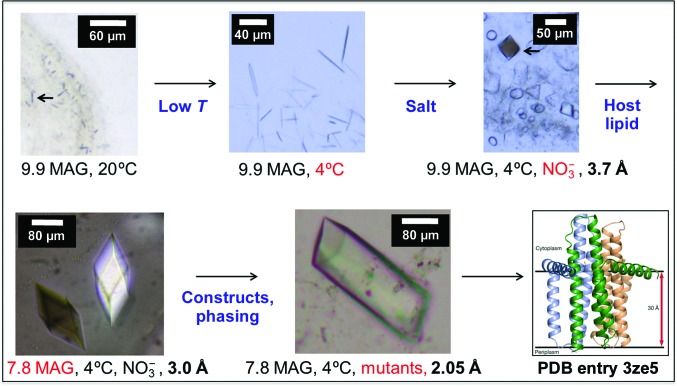
Critical steps in determining the *in meso* crystal structure of diacylglycerol kinase (DgkA). Diffracting crystals were obtained following extensive temperature, salt and host lipid screening (Li, Shah *et al.*, 2013 ▶). Experimental phasing proved challenging, but finally yielded a structure to a resolution of 2.05 Å (Li *et al.*, 2015 ▶).

The *in meso* method burst onto the scene almost two decades ago (Fig. 2 ▶). It was received with great anticipation for what it would deliver; perhaps it was to be the panacea. However, output in the early years was limited to naturally abundant, bacterial α-helical proteins bedecked with stabilizing and highly coloured prosthetic groups. The perceived restricted range, coupled with the challenges associated with handling the sticky and viscous cubic mesophase, meant that subsequent interest in the method waned. This was countered to some degree with the introduction of the *in meso* robot, a growing understanding of how the method worked at a molecular level and a continued demonstration of the general applicability of the method. However, interest in the method has rocketed of late with the success it has had, particularly in the GPCR field (Fig. 1 ▶, Table 1 ▶).

Improvements are needed for the method to thrive and for its longevity. Critically, the specialized materials and supplies upon which the method relies must be made more generally available and the method itself must be made more user-friendly and routine. New and improved *in meso* robots available on the market are tackling the user-friendliness issue. Workshops that involve hands-on demonstrations contribute to making the method more accessible. The author has been active in this area for past decade, with the latest workshop being held as part of the ICCBM15 meeting in Hamburg in September 2014 (http://www.iccbm15.org). There, 72 students were trained in the practicalities and finer details of *in meso* crystallo­genesis during the course of a three-day workshop (http://www.iccbm15.org/iccbm15_Workshop.xhtml). Online, open-access video demonstrations of the method are available (Caffrey & Porter, 2010 ▶; Li, Boland, Aragão *et al.*, 2012 ▶; Li, Boland, Walsh *et al.*, 2012 ▶).

Developments are needed in the area of crystal identification. Optical clarity is of the highest quality with the glass sandwich plates currently in use and this provides the ready detection of colourless, micrometre-sized crystals in normal light and between crossed polarizers with a light microscope. Detection by UV fluorescence is particularly powerful and convenient for tryptophan-containing proteins. Fluorescence labelling (Forsythe *et al.*, 2006 ▶) is also a route worth considering for the sensitive detection of early hits. Second-order nonlinear optical imaging of chiral crystals (SONICC) has been shown to sensitively and selectively detect certain membrane-protein crystals growing *in meso* (Kissick *et al.*, 2010 ▶).

Recovering crystals from the mesophase for data collection is a nontrivial undertaking (Caffrey & Cherezov, 2009 ▶; Li, Boland, Aragão *et al.*, 2012 ▶). This is especially true when harvesting is performed directly from glass sandwich plates. Typically, a glass cutter is used to open the well and to expose the mesophase. Teasing out and harvesting the crystal for immediate diffraction or snap-cooling in liquid nitrogen is most conveniently performed with a cryo-loop. Harvesting is a slow, painstaking, inefficient and cumbersome process, especially if it must be performed in a cold room and/or in subdued light. This whole area of harvesting calls out for innovation.

Data collection at a synchrotron is not exactly straightforward either. Given that *in meso*-grown crystals tend to be small, a micrometre-sized X-ray beam is required. Oftentimes, the crystal of interest is hidden from view in a bolus of opaque mesophase at 100 K on the cryo-loop. This means that locating the crystal and centring it requires rounds of diffraction rastering with a beam of progressively smaller size (Cherezov *et al.*, 2009 ▶). This same approach is used to advantage in finding the best diffracting part of a crystal. Locating crystals and centring based on X-ray fluorescence from heavy atoms in the sample is another option (Stepanov *et al.*, 2011 ▶). Effective and efficient diffraction rastering is now recognized as an important feature of the most up-to-date MX beamlines at synchrotron facilities worldwide, and steady improvements in the rastering process are being made. *In situ* screening and data collection are other areas that are under vigorous investigation (Bingel-Erlenmeyer *et al.*, 2011 ▶; Axford *et al.*, 2012 ▶). The *in situ* approach will benefit from improvements in sample presentation, high-performance gonio­meters, higher X-ray fluence, smaller and more stable beams, and faster detectors. Also, in the interests of the environment, cost, time and convenience, remote screening and data collection that is as easy and as efficient as it is on-site must be implemented.

Seeding has been used to advantage, especially with soluble proteins, to enable the production of structure-grade crystals in recalcitrant systems. Indeed, the recently introduced matrix seeding is proving to be particularly successful (D’Arcy *et al.*, 2014 ▶). A seeding protocol that is applicable to *in meso* crystallization would certainly be well received. Issues that need to be resolved include establishing that seeding actually works *in meso* and, if it does, the type of seed crystals and conditions that are most effective. Must seed crystals be grown *in meso* or can they be provided from alternative crystallization sources? It may be that seed crystals generated *in meso* could be used for crystallization trials *in surfo*. We have established that the bilayer of the cubic phase is fusogenic (Caffrey, 2008 ▶, 2009 ▶). Therefore, combining two boluses of mesophase, one with seed crystals and the other with target protein reconstituted under conditions that place it in the so-called nucleation zone, should in principle provide the conditions for seed-induced crystal growth. This area is deserving of further study.

Without exception, all 192 records in the PDB that refer to *in meso* structures exhibit layered or type I crystal packing. Whilst alternative packing arrangements are theoretically possible (§[Sec sec2]2), for the moment a very reasonable assumption is that type I packing prevails. If so, then it occurs to the author that this layered packing information might be used to advantage to solve *in meso* crystal structures. However, demonstrating that this is in fact useful ‘prior knowledge’ and that it can be exploited must be left to a suitably skilled and motivated crystallographer.

Given that membrane proteins are important drug targets, obtaining high-resolution crystal structures of target proteins with ligands bound is an important goal. Ligands often have limited water solubility. The bilayer of the mesophase can be used to advantage here as a reservoir from which ligands are provided by way of the bilayer itself or the aqueous compartments of the mesophase (see Li *et al.*, 2015 ▶). This same approach is also worth exploring with water-soluble proteins that we know can be crystallized *in meso* and with poorly soluble ligands made available at high concentrations by way of a surrounding, very local bilayer.

Finally, the method should begin to be used with really large proteins and complexes. The sponge phase (Cherezov, Clogston *et al.*, 2006 ▶), with its open aqueous channels and flatter, less rigid bilayer, should prove particularly useful in this regard. Using it in combination with novel hosting and additive lipid screens (Cherezov, Clogston *et al.*, 2006 ▶; Li *et al.*, 2011 ▶, 2014 ▶; Li, Shah *et al.*, 2013 ▶) will go a long way towards producing crystals and ultimately high-resolution structures where interactions that are integral to human health are revealed.

## Figures and Tables

**Table 1:** Integral membrane proteins and peptides crystallized by the *in meso* method that have yielded structures The table is current as of 26 September 2014. The total PDB record count is 192. As noted in [Sec sec18]18, as of September 2014 a ‘Search’ of ‘Everything’ from the PDB homepage (http://www.pdb.org) under ‘lipidic cubic phase’ yields 113 records and all are relevant. However, a search under ‘lipid cubic phase’ yields only 89 records. To recover all relevant *in meso* records we found it necessary to use ‘lipid* cubic phase*’ in combination with ‘sponge’, ‘lipdic’, ‘qubic’, ‘mesophase*’, ‘in *meso* phase’ and ‘LCP’ in a Text Search under the Advanced Search Interface on the PDB website.

Type	Name (PDB record count)	Organism	Function	Host and additive lipids	PDB entry (resolution, )
-Helical	GPCR (54)	*Homo sapiens*, *Rattus norvegicus*, *Mus musculus*, *Meleagris gallopavo *	G protein-coupled receptor	9.9 MAG + cholesterol; 7.7 MAG + cholesterol; 9.9 MAG	4phu (2.33), 3eml (2.60), 4eiy (1.80), 3qak (2.70), 4gbr (3.99), 2rh1 (2.40), 3d4s (2.80), 3ny9 (2.84), 3ny8 (2.84), 3nya (3.16), 3pds (3.50), 3p0g (3.50), 3odu (2.50), 3oe0 (2.90), 3oe6 (3.20), 3oe8 (3.10), 3oe9 (3.10), 4k5y (2.98), 3pbl (2.89), 4oo9 (2.60), 3rze (3.10), 3uon (3.00), 4mqs (3.50), 4mqt (3.70), 3vw7 (2.20), 4jkv (2.45), 4o9r (3.20), 4n4w (2.80), 4qim (2.61), 4qin (2.06), 3v2w (3.35), 3v2y (2.80), 4djh (2.90), 4lde (2.79), 4ldl (3.10), 4ldo (3.20), 4qkx (3.30), 4iaq (2.80), 4iar (2.70), 4ib4 (2.70), 4nc3 (2.80), 4n6h (1.80), 4l6r (3.30), 4ntj (2.62), 4pxz (2.50), 4py0 (3.10), 4ea3 (3.01), 4or2 (2.80), 4mbs (2.71), 4daj (3.40), 4grv (2.80), 4dkl (2.80), 4ej4 (3.40), 4bvn (2.10)
	Bacteriorhodopsin (39)	*Halobacterium salinarum*	Rhodopsin, nonvisual	9.9 MAG; -XylOC_16+4_; 95% monomethyl-DOPE, 5% DOPE-mPEG350	1ap9 (2.35), 1brx (2.30), 1qhj (1.90), 1c3w (1.55), 1c8r (1.80), 1c8s (2.00), 1cwq (2.25), 1qko (2.10), 1qkp (2.10), 1f4z (1.80), 1f50 (1.70), 1e0p (2.10), 1jv6 (2.00), 1jv7 (2.25), 1kg8 (2.00), 1kg9 (1.81), 1kgb (1.65), 1m0k (1.43), 1m0l (1.47), 1m0m (1.43), 1o0a (1.62), 1mgy (2.00), 1p8h (1.52), 1p8i (1.86), 1p8u (1.62), 1vjm (2.30), 1s8j (2.30), 1s8l (2.30), 2i1x (2.00), 2i20 (2.08), 2i21 (1.84), 2ntu (1.53), 2ntw (1.53), 2wjk (2.30), 2wjl (2.15), 3mbv (2.00), 3ns0 (1.78), 3nsb (1.78), 4fpd (2.65)
	Cytochrome *ba*_3_ oxidase (13)	*Thermus thermophilus*	Cytochrome oxidase	9.9 MAG	3s8f (1.80), 3s8g (1.80), 4fa7 (2.50), 4faa (2.80), 4gp4 (2.80), 4gp5 (2.70), 4gp8 (2.80), 4g7r (3.05), 4g70 (2.60), 4g71 (2.90), 4g72 (3.19), 4g7q (2.60), 4g7s (2.00)
	Diacylglycerol kinase (7)	*Escherichia coli* K-12	Enzyme	7.8 MAG; 7.9 MAG	3ze3 (2.05), 3ze4 (3.70), 3ze5 (3.10), 4bpd (3.30), 4brb (2.55), 4brr (2.44), 4d2e (2.28)
	MATE transporters (7)	*Pyrococcus furiosus*	Transporter	9.9 MAG	3vvn (2.40), 3vvo (2.50), 3vvp (2.91), 3vvr (3.00), 3vvs (2.60), 3w4t (2.10), 3wbn (2.45)
	Photosynthetic reaction centre (6)	*Blastochloris viridis*	Reaction centre	9.9 MAG	2wjm (1.95), 2wjn (1.86), 2x5u (3.00), 2x5v (3.00), 4ac5 (8.2), 4cas (3.50)
	Sensory rhodopsin II (6)	*Natronomonas pharaonis*	Rhodopsin, nonvisual	9.9 MAG	1jgj (2.40), 1gu8 (2.27), 1gue (2.27), 1h68 (2.10), 3qap (1.90), 3qdc (2.50)
	Photosynthetic reaction centre (5)	*Rhodobacter sphaeroides*	Reaction centre	9.9 MAG	1ogv (2.35), 2bnp (2.70), 2bns (2.50), 2gnu (2.00), 4tqq (2.50)
	Peptide (POT) transporter (5)	*Geobacillus kaustophilus*	Transporter	9.9 MAG	4ikv (1.90), 4ikw (2.00), 4ikx (2.30), 4iky (2.10), 4ikz (2.40)
	CDP-alcohol phosphotranspherase (4)	*Archaeoglobus fulgidus*	Enzyme	9.9 MAG	4o6m (1.90), 4o6n (2.10), 4q7c (3.10), 4mnd (2.66)
	Sensory rhodopsin IItransducer complex (4)	*Natronomonas pharaonis*	Rhodopsin, nonvisual	11.7 MAG	1h2s (1.93), 2f93 (2.00), 2f95 (2.20), 4gyc (2.05)
	Halorhodopsin (3)	*Halobacterium salinarum*	Rhodopsin, nonvisual	9.9 MAG	1e12 (1.80), 2jag (1.93), 2jaf (1.70)
	Peptide (POT) transporter (3)	*Streptococcus thermophilus*	Transporter	7.8 MAG	4d2b (2.35), 4d2c (2.47), 4d2d (2.52)
	Na^+^/bile acid symporter (2)	*Yersinia frederiksenii*	Transporter	9.9 MAG	4n7w (2.80), 4n7x (1.95)
	Sugar (SWEET) transporter (2)	*Leptospira biflexa*, *Vibrio* sp.	Transporter	9.9 MAG	4qnc (2.40), 4qnd (1.70)
	Protein insertase (YidC) (2)	*Bacillus halodurans*	Insertase	9.9 MAG	3wo6 (2.40), 3wo7 (2.20)
	K^+^ channel (2)	*Listeria monocytogenes*	Channel	9.9 MAG	4h33 (3.10), 4h37 (3.35)
	Sensory rhodopsin (2)	*Nostoc.* sp	Rhodopsin, nonvisual	9.9 MAG	1xio (2.00), 4tl3 (2.30)
	Channelrhodopsin (1)	*Chlamydomonas reinhardtii*	Rhodopsin, nonvisual	9.9 MAG	3ug9 (2.30)
	*Acetabularia* rhodopsin II (1)	*Acetabularia acetabulum*	Rhodopsin, nonvisual	9.9 MAG + cholesterol	3am6 (3.20)
	Proteorhodopsin (1)	*Exiguobacterium sibiricum*	Rhodopsin, nonvisual	9.9 MAG	4hyj (2.30)
	Light-harvesting complex II (1)	*Rhodoblastus acidophilus*	Light-harvesting complex II	9.9 MAG	2fkw (2.45)
	Cytochrome *caa*_3_ oxidase (1)	*Thermus thermophilus*	Cytochrome oxidase	7.7 MAG	2yev (2.36)
	Prostaglandin E2 synthase 1 (1)	*Homo sapiens*	Enzyme	8.8 MAG + DOPC	4bpm (2.08)
	Na^+^/Ca^+^ exchanger (1)	*Methanocaldococcus jannaschii*	Exchanger	9.9 MAG	3v5u (1.90)
	Ca^2+^/H^+^ exchanger (VCX1) (1)	*Saccharomyces cerevisiae*	Exchanger	9.9 MAG	4k1c (2.30)
	H^+^/Ca^2+^ exchanger (1)	*Archaeoglobus fulgidus*	Exchanger	9.9 MAG	4kpp (2.30)
	Na^+^ symporter MhsT (1)	*Bacillus halodurans*	Symporter	7.8 MAG	4us4 (2.60)
	Claudin (1)	*Mus musculus*	Junction protein	9.9 MAG	4p79 (2.40)
	GPCRG protein complex (1)	*Bos taurus*, *Rattus norvegicus*, *Homo sapiens*	G protein-coupled receptorG protein complex	7.7 MAG + cholesterol	3sn6 (3.20)
-Barrel	AlgE (3)	*Pseudomonas aeruginosa*	Transporter	7.8 MAG	4afk (1.90), 4azl (2.80), 4b61 (2.40)
	OmpF (3)	*Escherichia coli*	Channel	9.9 MAG	3poq (1.90), 3pou (2.80), 3pox (2.00)
	Vitamin B_12_ transporter ButB (1)	*Escherichia coli*	Transporter	9.9 MAG	2guf (1.95)
	Adhesin/invasin OpcA (1)	*Neisseria meningitidis*	Adhesin	9.9 MAG	2vdf (1.95)
	Intimin (1)	*Escherichia coli*	Adhesin	9.9 MAG	4e1s (1.86)
	Invasin (1)	*Yersinia pseudotuberculosis*	Adhesin	9.9 MAG	4e1t (2.26)
-Helix	Gramicidin D (4)	*Brevibacillus brevis*	Channel	7.7 MAG; 8.8 MAG; 11.7 MAG; 9.9 MAG	2y5m (1.08), 2y6n (1.26), 3zq8 (1.70), 2xdc (1.70)
